# Usage and cost of first-line drugs for patients referred to inpatient anthroposophic integrative care or inpatient conventional care for stress-related mental disorders—a register based study

**DOI:** 10.1186/s12906-015-0865-3

**Published:** 2015-10-14

**Authors:** Tobias Sundberg, Laith Hussain-Alkhateeb, Torkel Falkenberg

**Affiliations:** Department of Neurobiology Care Sciences and Society, Division of Nursing, Research Group Integrative Care, Karolinska Institutet, Huddinge, Sweden; I C – The Integrative Care Science Center, Järna, Sweden; Health Metrics, Sahlgrenska Academy, Institute of Medicine, University of Gothenburg, Gothenburg, Sweden; Prevalens i Väst AB, Göteborg, Sweden

## Abstract

**Background:**

Stress-related mental disorders (SRMD) are common and costly. Rehabilitation strategies, including pharmacotherapy, may be complicated to evaluate. Previous research has indicated increased quality of life and self-rated health for SRMD patients that receive a combination of conventional and complementary therapies, i.e. integrative care. The aim of this retrospective registry study was to explore and contrast the prescription of first-line drugs for SRMD patients referred to hospital inpatient anthroposophic integrative care (AIC) or inpatient conventional care (CC).

**Methods:**

SRMD patients that had received AIC or CC were identified through high-quality inpatient registry data from Stockholm County Council and matched by available background characteristics including diagnosis (ICD-10: F43), age, gender and socio-economics. General disease load was estimated by analysis of ICD-10 chapter data. The Swedish Prescribed Drug Register was then used to investigate purchased defined daily doses (DDD) and cost of drugs from 90-days before/after, and 180-days before/after, the first visits (index) to AIC and CC respectively. First-line drug categories were Anatomical Therapeutic Chemical classification codes N05A (antipsychotics), N05B (anxiolytics), N05C (hypnotics and sedatives) and N06A (antidepressants).

**Results:**

There were no statistically significant differences between the AIC (*n* = 161) and the CC (*n* = 1571) cohorts in terms of background characteristics and the overall disease loads were similar between the groups the preceding year. At baseline, the prescription of first-line anxiolytics and antidepressants were not statistically different between groups whereas the prescription of antipsychotics and hypnotics/sedatives were lower for the AIC cohort. The overall change in drug prescriptions and costs during the investigated periods, both for the 90-days before/after and for the 180-days before/after the index visit, showed a general decrease within the AIC cohort with significantly less prescribed anxiolytics and hypnotics/sedatives. During the same time periods there was a general increase in prescriptions and costs of first-line drugs within the CC cohort. The overall disease loads were generally stable within both cohorts over time, except that the CC cohort had increased visits registered with an ICD-10 F-chapter diagnosis the year after index.

**Conclusions:**

The results suggests that there may be different drug utilization patterns for SRMD patients referred to AIC or CC. Different management strategies between AIC and CC providers, different SRMD disease severities and different preferences of patients referred to AIC and CC are hypothetical differentiating factors that may influence drug outcomes over time. Additional studies including prospective and randomized clinical trials are warranted to determine if there is a causal link between inpatient AIC and reduced drug utilization.

## Background

### Mental ill health

The individual and societal burdens of mental ill health, including stress related mental disorders (SRMD), are significant. The concept of SRMD, referring to code F43 in the International Classification of Diseases version 10 (ICD-10), involves short- and long-term reactions to severe stress including post-traumatic stress disorders, adjustment disorders with depressive reactions and other reactions to severe stress such as exhaustion disorder or other emotional disturbances [1, 2].

Evidence of the heavy burden of mental ill health in Sweden can be found in the increase and predominance of psychological disorders among people with sickness absence and those receiving compensation for sickness or activity loss [3, 4]. For new sickness and activity compensation in Sweden in 2012, psychological disorders constituted the most prevalent diagnostic group among both women and men across almost every age group, ranging from 25 to 30 % among those granted compensation in the oldest age group, up to 85 % in the youngest age group [3].

The Swedish National Board of Health and Welfare has estimated that 20-40 % of the population suffer from mental ill health, ranging from mild to severe disorders [5]. The prevalence of SRMD varies among different disorders and contexts. In Sweden e.g. the life-time prevalence of post-traumatic stress disorder in the general population has been estimated at about 6 %, with twice as many women as men affected [6]. Similarly, prevalence figures of up to 20 % have been reported for perceived exhaustion and related symptoms [5, 7, 8], with an estimated ratio of 1:2 for self-reported exhaustion among men and women respectively [9].

### Costs

It is difficult to estimate the specific costs for SRMD alone compared to other psychiatric diagnoses. The annual cost of psychiatric disorders in Sweden has been estimated at euro (EUR) 9.4 billion, where the sum of direct costs, including the cost of drugs, comprises approximately 20 % of the total costs [10]. Specifically, regarding first-line drugs targeting psychiatric disorders, defined as pharmacy sales price excluding VAT, it has been estimated that antidepressants comprise almost 60 % of the total drug costs followed by antipsychotics, hypnotics, sedatives and anxiolytics [10]. The Swedish Dental and Pharmaceutical Benefits Agency (TLV), a central government agency that determines whether a pharmaceutical product shall be subsidized by the state, has specifically investigated the use of antidepressants, i.e. the ATC-N06A drug group, in terms of costs and effects [11]. In short, the TLV reported that 700 000 people were treated with antidepressants in 2007; that the associated costs for antidepressant pharmacotherapy was high; that less than every second patient reached a satisfactory treatment result from the initial antidepressant treatment; that adverse drug events are problematic and may contribute to patients not being able to fulfil or continue their treatments; and that more effective drugs and more knowledge how to use these drugs are needed [11]. TLV concludes that there are gaps in the evidence in healthcare to make decisions about effective treatment – not least to put antidepressant drugs in relation to other forms of treatment – and that more knowledge about these drugs is needed for patients to get effective treatment [11].

### Managing SRMD

Conclusive evidence-based clinical guidelines for managing SRMD in Sweden are currently lacking [2]. Hence, conventional management of SRMD is mainly consensus-based and generally recommends multi-modal and multi-professional rehabilitation, which may include psychological and group counselling, individually tailored stress reduction methods alongside support for lifestyle changes and workplace interaction [2, 12, 13]. Such rehabilitation strategies may be complemented by pharmacotherapy for anxiety, sleeping problems and depressive reactions, e.g. using anxiolytics, sedatives and antidepressants [13]. Pharmacotherapy with anxiolytics and sedatives should generally be administered at low doses and used infrequently as needed [13]. Antidepressants, if indicated, are typically prescribed in the long term with follow-ups after 1, 6 and 12 months [13]. Sick-leave or physical activity can also be part of the rehabilitation plan [13].

Integrative care aims to adhere to the same high standard of services as those provided by conventional care, with the addition of integrating selected complementary therapies. The objective is to provide patient-centred, personalised, safe and effective health care with as few side effects as possible [14]. Integrated health services are increasingly becoming part of international and national health care policy and research agendas [15–19], and recommendations for integrative care strategies can be found in national care guidelines relating to mental and physical disorders such as depression and chronic pain whereby for example mindfulness, massage and manual therapy may be included in the care for certain diagnoses [20, 21].

Anthroposophic integrative care (AIC) is a well-established form of integrative health service provision provided by conventionally trained medical doctors, nurses and therapists that have additional training in the practice and integration of selected complementary therapies such as massage, natural remedies, art, music and movement therapies together with conventional therapies [22]. Recently, the Swiss government reported primarily positive outcomes, few risks, good tolerability and favourable cost structure, together with high patient satisfaction of AIC [23]. Similarly, Swedish research has indicated improved patient reported outcome measures of quality of life and self-rated health, following AIC for SRMD [24] and that pain patients that have received AIC may reduce their need of analgesics [25]. AIC is implemented in numerous hospitals and medical practices internationally [23, 26]. In Sweden, patients have been referred to AIC rehabilitation at Vidarkliniken, the largest Scandinavian anrthroposophic hospital, since the 1980s [22, 27]. The majority of large county councils in Sweden have caregiving agreements with Vidarkliniken that allow for referrals of SRMD patients to inpatient AIC on similar terms as referrals of SRMD patients to inpatient conventional care (CC). Referral of SRMD patients to inpatient care, whether AIC or CC, are typically managed via general practitioners in primary care.

Given the common practice of integrative care in private and public health settings, scientific studies investigating different health care models are needed to inform decision-making and health sector reform. The randomised placebo controlled trial is the gold standard for the evaluation of specific efficacy, for instance of newly regulated drugs. Comparative effectiveness research, including pragmatic clinical trials and registry evaluations, where different interventions are evaluated, compared and contrasted under normal clinical circumstances, is an evaluation approach that is increasingly recommended by Health Technology Assessment boards in the strive for more valid and generalizable outcomes to clinical health care provision [28–31]. Sweden has comprehensive population based health registries accessible to researchers, which makes registry studies in Sweden a valid and cost-effective method of research. The aim of this study was to explore and contrast the prescription of first-line drugs for SRMD patients referred to hospital inpatient AIC or inpatient CC.

## Methods

### Design and setting

Retrospective registry study, Stockholm County, Sweden.

### Patient observations and matching

Patients with SRMD that had been referred to the largest inpatient AIC hospital in Sweden (Vidarkliniken) were identified through the high-quality inpatient registry (patientregistret för slutenvård) of Stockholm County Council for the period 2005–2010. The same registry and periods were used to identify SRMD patients that had been referred to inpatient CC, which were matched to the AIC patients by available background characteristics including:

- diagnosis (International Classification of Diseases, version 10: code F43),
- age at index visit (18–39 years, 40–49 years, 50–59 years, and 60+),
- gender (male/female) and
- socio-economic group (three classes: High, Affluence and Low).

The latter was achieved by utilizing the Mosaic geo-demographic segmentation system employed by Stockholm County Council [32]. The Mosaic system makes use of a multivariate statistical classification technique for categorizing the population into different socio-economic groups [33]. It relies mainly on data from Statistics Sweden with its high-quality population registries, including the Swedish Population Register, the Real Estate Tax Register, the Income and Wealth Register, the Register of Education and data from parliamentary elections [33, 34]. There are thousands of Mosaic geographic areas in Sweden, which implies high accuracy without the risk of detrimental effects on the integrity of individual data protection [33]. Based on the available subgroup sizes for matching, the patients of the CC cohort were randomly sampled in a 1:10 proportion to AIC patients to ensure a balanced sample representative of the totality of provided CC.

### Index visit, target diagnosis and care provision

The index visit was defined as the first registered inpatient visit to AIC or CC with the target SRMD diagnosis (ICD-10: F43), which by description entails reaction to severe stress and adjustment disorders, i.e. referring to long- or short-term reactions caused by exposure to severe stress. The F43 diagnosis class accommodates acute and post-traumatic stress disorders, adjustment disorders – with brief or extended depressive reactions, anxiety or other emotional disturbances – and other reactions to severe stress such as exhaustion disorder [1, 2]. Patients referred to inpatient CC did not received inpatient AIC, and patients referred to inpatient AIC had not previously been registered for inpatient CC for the same target diagnosis. Licensed caregivers providing inpatient AIC in Sweden typically have dual proficiency, which means they are trained in both conventional therapies (e.g. medicine or nursing) and anthroposophic complementary therapies (e.g. natural remedies, massage, art and music therapy). Thus, Swedish inpatient AIC provision typically consists of a personalized integration of conventional and anthroposophic complementary therapies.

### Outcomes and data collection

The primary outcomes were:

i. the changes in prescribed and sold defined daily doses (DDD)—a term referring to the assumed average maintenance dose per day for a drug used for its main indication [35]; and
ii. the associated costs of the prescribed drugs.

The study outcomes were investigated over 180-days and 360-days time periods, i.e. the data for 90 days before/after and for 180 days before/after, the index visits to AIC or CC, respectively. Data on drug prescriptions, purchased DDD and costs in Swedish kronor were collected for 2005 to 2010 from the Swedish Prescribed Drug Register, which is operated and controlled by the National Board of Health and Welfare (Socialstyrelsen), a Swedish government agency under the Ministry of Health and Social Affairs [36]. The cost of drugs was converted from Swedish kronor to EUR using the average exchange rate during 2013 corresponding to nine Swedish kronor to one EUR. All costs were expressed in 2013 year’s prices using the Swedish consumer price index for recalculations as necessary. The selected drug groups and combinations were restricted to those covered by the Anatomical Therapeutic Chemical classification system [37], i.e. codes N05A (antipsychotics), N05B (anxiolytics), N05C (hypnotics and sedatives) and N06A (antidepressants), which includes codes relevant to first-line drugs recommended in the management of SRMD in Sweden [13]. Up to four substrings were applied to define the selected ATC groups. The derived DDD rate per patient, per day and per period, was based on the number of AIC and CC patients in the study sample.

General disease load/co-morbidity was estimated by frequency and mean value analysis of ICD-10 chapter data (A-Z) based on registered inpatient visits 365-days before and after the first inpatient visits for ICD-10: F43 (index). The target SRMD diagnosis ICD-10: F43 was included in chapter F that details “Mental and behavioural disorders”. Differences of derived frequencies per diagnostic category were calculated between the cohorts by using the attributable difference as a simple and reliable approach.

### Statistical analysis

Standard statistical procedures were employed for calculating means, standard deviations and 95 % confidence intervals. Data was initially tested for normal distribution and as some violation was detected, mainly in cost data, these were submitted to both parametric and non-parametric testing procedures. However, as satisfaction with the required criteria for normality was partially met, parametric tests were deemed most appropriate to provide generalizable findings. Although this study relies on parametric methods to generate conclusions, non-parametric Wilcoxon rank sum testing was also employed to provide sensitivity testing for robustness and reliability of results. While Chi-square test was used for the categorical background variables, paired and unpaired t-tests were performed to test for differences between means of continuous variables, i.e. the DDDs and costs of drugs for the cohorts over time. Differences in disease load were tested at baseline and follow-up periods by t-test and Poisson regression methods. All statistical tests were two-tailed and corrected for unequal group variances as necessary. Cohen’s d was used to estimate the effect sizes with 95 % CI between the cohorts, ranging from small (d = 0.2), to medium (d = 0.5), to large (d = 0.8) effect sizes [38]. The level of statistical significance was 5 % (two-tailed test). Statistical software included SAS and STATA13.

### Ethics statement

The study was approved by the Regional Ethics Committee in Stockholm. Data was retrieved as de-identified/anonymized files from the national and county council health care registries, thus no written informed consent procedures were conducted. Results were only analysed and reported at group level.

## Results

**Fig. 1:**
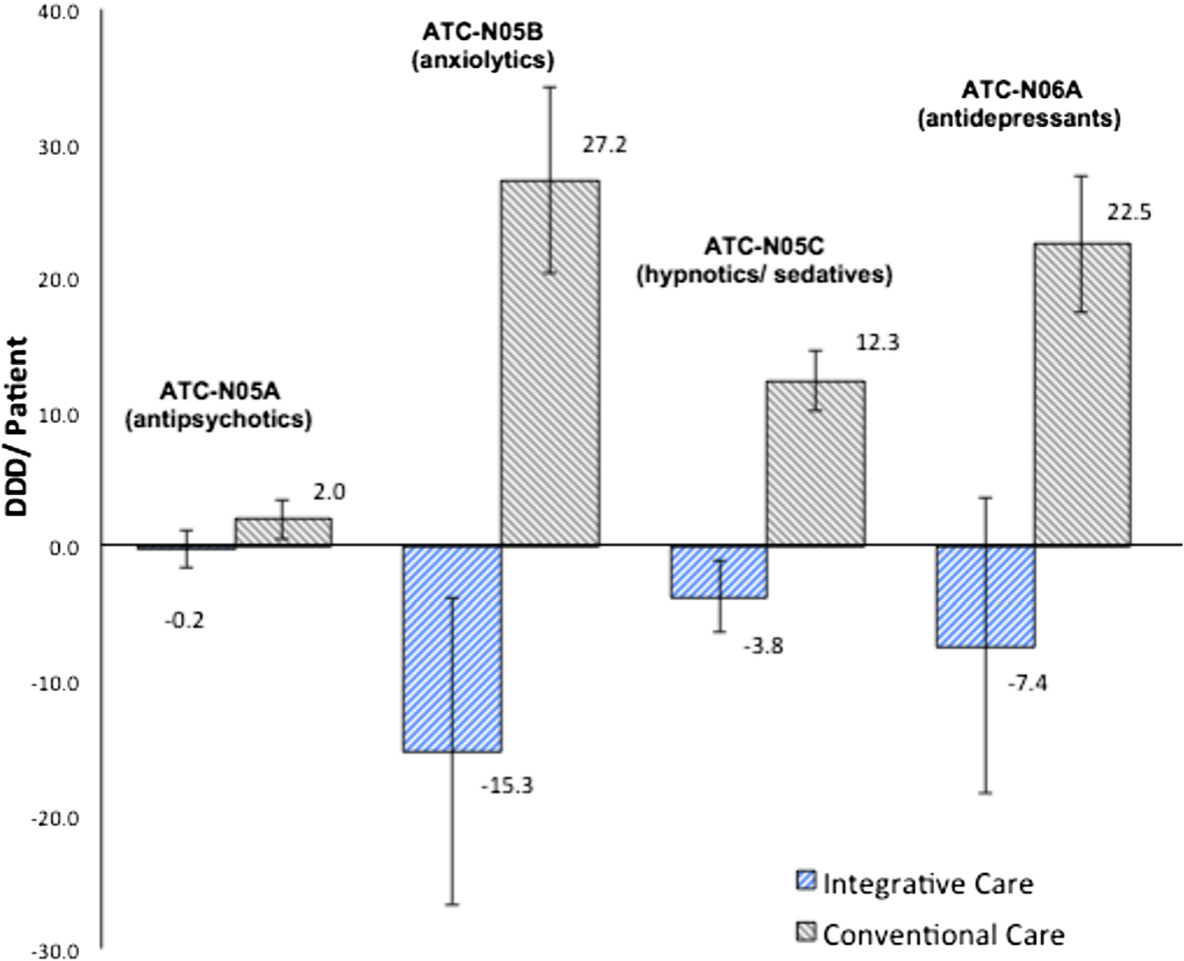
Change in drug prescriptions from 180-days before to 180-days after first observed inpatient visit with diagnosis F43 within the integrative care and conventional care cohorts respectively. DDD, Defined daily dose. Drug categories according to the Anatomical Therapeutic Chemical (ATC) classification system

There were no statistically significant differences between the matched AIC (*n* = 161) and CC (*n* = 1571) cohorts in terms of observable background characteristics (Table 1) and the cohorts had similar overall disease loads the year preceding the index visit (Table 5). The prescription of first-line anxiolytics and antidepressants were not statistically different between groups at baseline, whereas the prescription of antipsychotics and hypnotics/sedatives were significantly lower for the AIC cohort both at the 90 and 180 days pre-index visit periods (Table 2). The subsequent changes of drug prescriptions and costs during the investigated periods, i.e. 90-days before/after and 180-days before/after the index visit, showed a general decrease within the AIC cohort with significantly less prescribed anxiolytics and hypnotics/sedatives (Tables 3 and 4). Within the CC cohort there was an observed general increase of prescriptions and costs of first-line drugs during the same time periods (Tables 3 and 4). Figure 1 contrasts the changes of drug utilisation within the AIC and the CC cohorts over time.

**Table 1:** Characteristics of the integrative care and conventional care patient cohorts

	Integrative Care (*N* = 161)	Conventional Care (*N* = 1571)	Statistical difference (*P*-value)
	% (n)	% (n)	
Gender			0.927
Female	91.3 (147)	91.1 (1431)	
Male	8.7 (14)	8.9 (140)	
Age			0.995
0–39 years	19.9 (32)	19.1 (300)	
40–49 years	38.5 (62)	39.2 (615)	
50–59 years	29.2 (47)	29.0 (456)	
60+ years	12.4 (20)	12.7 (200)	
Socio-economic classification (Mosaic)			0.953
High	50.9 (82)	52.2 (820)	
Affluent	17.4 (28)	18.5 (290)	
Low	29.8 (48)	29.3 (461)	
Missing	1.9 (3)	0	

All patients were diagnosed with code F43 according to the International Classification of Diseases version 10. Analyses by Chi-square test

**Table 2:** Drug prescriptions and cost of drugs 90 and 180 days before the first observed inpatient visit with diagnosis F43 for the integrative care and conventional care cohorts

	Integrative Care (*N* = 161)	Conventional Care (*N* = 1571)	Statistical difference (*P*-value)
DDD/patient			
ATC-N05A 90-days before	0.8 (−0.1 to 1.7)	2.9 (2.1 to 3.8)	<0.001
ATC-N05B 90-days before	26.5 (16.6 to 36.3)	32.3 (27.9 to 36.7)	0.287
ATC-N05C 90-days before	5.7 (3.5 to 7.9)	11.9 (10.5 to 13.3)	<0.001
ATC-N06A 90-days before	16.6 (9.6 to 23.7)	21.0 (18.2 to 32.8)	0.260
ATC-N05A 180 days before	1.3 (−0.01 to 2.7)	5.1 (3.7 to 6.5)	<0.001
ATC-N05B 180 days before	41.4 (25.8 to 57.1)	54.2 (46.4 to 61.9)	0.150
ATC-N05C 180 days before	10.7 (6.9 to 14.5)	20.6 (18.1 to 23.0)	<0.001
ATC-N06A 180 days before	33.5 (20.1 to 46.7)	37.3 (32.6 to 42.0)	0.592
Cost/patient (EUR)			
ATC-N05A 90-days before	5.4 (−0.3 to 11.1)	19.4 (13.8 to 24.9)	<0.001
ATC-N05B 90-days before	4.8 (2.9 to 6.6)	6.4 (5.5 to 7.2)	0.133
ATC-N05C 90-days before	4.9 (3.0 to 6.8)	8.4 (7.4 to 9.4)	0.002
ATC-N06A 90-days before	27.5 (14.6 to 40.4)	24.8 (21.3 to 28.2)	0.699
ATC-N05A 180 days before	6.8 (−0.3 to 13.9)	36.5 (26.9 to 46.2)	<0.001
ATC-N05B 180 days before	7.6 (4.5 to 10.7)	11.4 (9.8 to 13.0)	0.036
ATC-N05C 180 days before	8.2 (5.3 to 11.2)	14.7 (12.8 to 16.5)	<0.001
ATC-N06A 180 days before	50.3 (28.5 to 72.1)	46.1 (40.3 to 51.9)	0.722

DDD, Defined daily dose. EUR, euro. Drug categories according to the Anatomical Therapeutic Chemical (ATC) classification system: N05A, antipsychotics; N05B, anxiolytics; N05C, hypnotics and sedatives; and N06A, antidepressants. Average (95 % confidence interval) values. Analyses by t-tests (two tailed)

**Table 3:** Change in drug prescriptions and cost of drugs during 90 days before/after the first observed inpatient visit with diagnosis F43 for the integrative care and conventional care cohorts

	Integrative Care (*N* = 161)	Conventional Care (*N* = 1571)	Conventional-Integrative	Conventional-Integrative
	Change from 90-days before to 90-days after index visit		Difference in change between groups	Effect Size (Cohen’s d)
DDDs/patient				
ATC-N05A	−0.3 (−1.1 to 0.6), *p* = 0.518	0.7 (−0.2 to 1.6), *p* = 0.147	1.0 (−0.3 to 1.5), *p* = 0.515	0.1 (−0.1 to 0.2)
ATC-N05B	−11.5 (−19.7 to −3.2), *p* = 0.003	16.9 (12.4 to 21.3), *p* < 0.001	28.3 (14.2 to 42.4), *p* < 0.001	0.3 (0.2 to 0.5)
ATC-N05C	−2.1 (−3.6 to −0.6), *p* = 0.004	6.8 (5.5 to 8.1), *p* < 0.001	8.9 (4.8 to 13.0), *p* < 0.001	0.4 (0.2 to 0.5)
ATC-N06A	−2.1 (−7.8 to 3.6), *p* = 0.473	10.4 (7.8 to 13.0), *p* < 0.001	12.5 (4.2 to 20.8), *p* < 0.001	0.2 (0.1 to 0.4)
Cost/patient (EUR)				
ATC-N05A	5.3 (−11.9 to 22.6), *p* = 0.542	14.5 (7.7 to 21.4), *p* < 0.001	9.2 (−12.9 to 31.3), *p* = 0.415	0.1 (−0.1 to 0.2)
ATC-N05B	−2.4 (−4.1 to −0.7), *p* = 0.003	3.2 (2.3 to 4.2), *p* < 0.001	5.6 (2.6 to 8.7), *p* < 0.001	0.3 (0.1 to 0.5)
ATC-N05C	−2.2 (−3.5 to −0.8), *p* = 0.001	2.9 (2.1 to 3.7), *p* < 0.001	5.1 (2.4 to 7.7), *p* < 0.001	0.3 (0.2 to 0.5)
ATC-N06A	−9.2 (−20.6 to 2.3), *p* = 0.058	13.0 (9.1 to 16.9), *p* < 0.001	22.2 (9.4 to 34.9), *p* < 0.001	0.3 (0.1 to 0.4)

DDD, Defined daily dose. EUR, euro. Drug categories according to the Anatomical Therapeutic Chemical (ATC) classification system: N05A, antipsychotics; N05B, anxiolytics; N05C, hypnotics and sedatives; and N06A, antidepressants. Average (95 % confidence interval), *p* values; Analyses by t-tests (two tailed). Cohen’s d: small 0.2, medium 0.5, large 0.8

**Table 4:** Change in drug prescriptions and cost of drugs during 180 days before/after first observed inpatient visit with diagnosis F43 for the integrative care and conventional care cohorts

	Integrative Care (N=161)	Conventional Care (N=1571)	Conventional-Integrative	Conventional-Integrative
	Change from 180-days before to 180-days after index visit		Difference in change between groups	Effect size (Cohen’s d)
DDDs/patient				
ATC-N05A	−0.2 (−1.6 to 1.2), *p* = 0.767	2.0 (0.5 to 3.5), *p* = 0.004	2.2 (−2.4 to 6.8), *p* = 0.351	0.1 (−0.1 to 0.2)
ATC-N05B	−15.3 (−26.7 to −3.8), *p* = 0.004	27.2 (20.3 to 34.1), *p* < 0.001	42.5 (20.6 to 64.4), *p* < 0.001	0.3 (0.2 to 0.5)
ATC-N05C	−3.8 (−6.4 to −1.2), *p* = 0.002	12.3 (10.1 to 14.5), *p* < 0.001	16.1 (9.2 to 22.9), *p* < 0.001	0.4 (0.2 to 0.5)
ATC-N06A	−7.4 (−18.4 to 3.5), *p* = 0.091	22.5 (17.4 to 27.5), *p* < 0.001	29.9 (13.7 to 46.1), *p* < 0.001	0.3 (0.1 to 0.5)
Cost/patient (EUR)				
ATC-N05A	12.8 (−13.9 to 39.5), *p* = 0.345	25.7 (14.8 to 36.5), *p* < 0.001	12.9 (−22.1 to 47.8), *p* = 0.471	0.1 (−0.1 to 0.2)
ATC-N05B	−2.8 (−4.7 to −0.8), *p* = 0.003	4.5 (3.0 to 6.0), *p* < 0.001	7.2 (2.5 to 12.0), *p* = 0.003	0.3 (0.1 to 0.4)
ATC-N05C	−2.7 (−4.6 to −0.9), *p* = 0.002	5.4 (3.9 to 6.9), *p* < 0.001	8.1 (3.5 to 12.7), *p* < 0.001	0.3 (0.1 to 0.5)
ATC-N06A	−12.6 (−31.1 to 5.9), *p* = 0.181	27.9 (21.3 to 34.6), *p* < 0.001	40.5 (18.9 to 62.1), *p* < 0.001	0.3 (0.1 to 0.5)

DDD, Defined daily dose. EUR, euro. Drug categories according to the Anatomical Therapeutic Chemical (ATC) classification system: N05A, antipsychotics; N05B, anxiolytics; N05C, hypnotics and sedatives; and N06A, antidepressants. Average (95 % confidence interval), *p* values; Analyses by t-tests (two tailed). Cohen’s d: small 0.2, medium 0.5, large 0.8

The effect sizes expressed as Cohen’s *d* ranged from small to medium (Tables 3 and 4). The drug prescriptions, expressed in DDD/1000 patients/day, were higher for the CC cohort computing an extra amount of: Anxiolytics 82.2, Antidepressants 19.7, Hypnotics and sedatives 17.4, for the 90 days period and; Anxiolytics 112.3, Antidepressants 60.3, Hypnotics and sedatives 31.4 for the 180 days period. Correspondingly, the costs of these drugs, expressed as cost/1000 patients/day, were higher for the CC cohort: Antidepressants EUR 65.4, Anxiolytics EUR 16.9, Hypnotics/sedatives EUR 15.5 (90-days period); and Antidepressants EUR 96.0, Anxiolytics EUR 20.3, Hypnotics/sedatives EUR 20.2 (180-days period).

The overall disease load based on all registered ICD-10 diagnostic chapters (A-Z) was similar at baseline and generally stable for both cohorts over time, with one notable exception, the CC cohort had increased visits with an ICD-10 F-chapter diagnosis, which covers mental and behavioural disorders, the year after the index visit (Table 5).

**Table 5:** Number of ICD-10 diagnostic categories (A-Z) per patient based on registered inpatient visits 365-days PRE/POST first visit for F43 (index visit)

	Integrative care (n 161)		Conventional care (n 1571)		Conventional-Integrative
	365 days PRE	Change 365 days POST	365 days PRE	Change 365 days POST	Attributable difference in change between groups
ICD-10 category					
A	0.05	0.02	0.06	0.02	0.00
B	0.13	−0.06	0.19	−0.04	0.02
C	0.24	0.30	0.31	0.14	−0.16
D	0.30	−0.16	0.19	0.02	0.18
E	0.28	0.13	0.41	0.03	−0.10
**F**	**5.94**	**−1.24**	**6.22**	**5.90**	**7.14**
G	0.27	−0.06	0.25	−0.01	0.05
H	0.27	−0.11	0.22	0.06	0.17
I	0.58	−0.16	0.52	0.05	0.21
J	0.53	−0.09	0.45	−0.05	0.04
K	0.36	−0.14	0.32	−0.02	0.11
L	0.20	0.02	0.18	0.01	−0.01
M	0.82	0.02	0.92	−0.02	−0.05
N	0.31	0.04	0.44	0.06	0.02
O	0.05	−0.02	0.13	−0.01	0.01
P	0.00	0.00	0.00	0.00	0.00
Q	0.01	0.00	0.02	0.00	0.00
R	0.99	−0.16	1.12	−0.08	0.09
S	0.30	−0.06	0.26	0.15	0.20
T	0.09	0.01	0.33	0.04	0.03
U	0.00	0.00	0.00	0.00	0.00
V	0.00	0.01	0.01	−0.01	−0.02
W	0.06	0.02	0.08	0.02	0.00
X	0.01	0.04	0.09	0.07	0.03
Y	0.01	0.03	0.06	0.00	−0.03
Z	1.56	0.18	1.69	0.31	0.13

ICD-10, International Classification of Diseases version 10. Main diagnostic category F (Mental and behavioural disorders) in bold

## Discussion

The current study set out to explore and contrast the prescription of first-line drugs for SRMD patients referred to hospital inpatient AIC or inpatient CC. The overall prescription pattern within each cohort showed a general decrease for the AIC cohort and an increase for the CC cohort. Notably, the retrospective study design doesn’t allow for any causative conclusions of the reasons for the observed trends. Accordingly, the study findings may rather be used for discussing and generating hypothesis of potential differentiating factors that may have influenced the drug outcomes within each cohort over time. Here multiple plausible contributing factors may be considered, e.g. different management strategies between AIC and CC providers, different SRMD disease severities and different preferences of patients referred to AIC and CC, as well as unknown confounders and selection bias, which may all have influenced the observed drug outcomes.

One hypothesis is that AIC management, by integrating complementary health approaches, may be able to help SRMD patients better cope with their disorder at the same time relying less on the use of prescription pharmaceuticals. Supporting this hypothesis is the increased health-related quality of life and improved and self-rated health that has been reported for SRMD patients receiving this type of AIC [24]. Similarly, emerging evidence of reduced utilisation of pharmaceuticals and health resources have been reported in previous studies of AIC and other types of integrative care, e.g. lower prescription rates and less adverse drug reactions relating to acute respiratory and ear infections [39], reduced use of analgesics for pain patients in inpatient [25] or primary care settings [40–42], and generally less use of health care resources associated with integrative care compared to conventional care [43].

On the other hand, it is currently unknown whether the observed drug prescription patterns reflect a management issue per se, e.g. that CC providers/patients may prioritize pharmacotherapy over other types of treatment compared to AIC providers/patients, or if the patients that were referred to CC actually had more severe SRMD that motivated more prescriptions of first-line drugs. Notably, despite the use of high-quality registries, the data did not contain information on duration, intensity or severity of disease. Hence, despite the similar profiles of the integrative and conventional cohorts at baseline, it is possible that conventional patients either already had, or developed, more severe SRMD over time and that the severity of disease may influence e.g. referral mechanisms or drug prescription patterns. The relative increase for CC patients in the frequency of registered inpatients visits in the ICD-10 F chapter for mental and behavioural disorders is an indication reflecting this possibility. However, the overall disease load was generally stable and similar within both cohorts over time, although the AIC cohort had somewhat decreased visits in the F chapter the year after the first visit. Thus, in that sense, the reduced usage of medication seems to not have negatively impacted on the disease load of the AIC patients. The relative decline in F-chapter disease burden for AIC patients observed in the current study may point towards potential cost savings with AIC for SRMD rehabilitation. However, it is important to acknowledge that the frequency of visits relating to diagnostic chapters of registered inpatient visits *per se* is not necessarily equal to a decrease in the use of health care resources. Importantly, factors such as total length of hospital stay, use of outpatient or specialist care and sick-leave, also need to be accounted for.

Drugs can be of tremendous benefit when properly prescribed and tolerated. However, if they are not rationally used, and for too long periods, and especially if there is inadequate scientific evidence to inform appropriate clinical integration of pharmacological care [44–46], the prospect of finding solutions to SRMD rehabilitation that rely less on the use of pharmacotherapy may be a worthwhile incentive. Clearly, improved rational use of drugs would result in fewer adverse drug reactions. It has been estimated that 15 % of all reported adverse drug events are caused by drugs in the Anatomical Therapeutic Chemical “N” category, where 60 % are caused by antipsychotic, anxiolytic, hypnotic and sedative and antidepressant drugs [47]. Close to 40 % of these reported adverse drug events were deemed serious, and over 40 % were unexpected [47]. Notably, the majority of the referred AIC patients where women, who generally may be affected to a greater extent by adverse drug reactions than men. Specified side effects range from nervous systems complaints such as headache, tremor and dizziness, over psychiatric conditions including insomnia and restlessness, to gastrointestinal problems with nausea and dryness of mouth, and increased weight [47]. Principally, reduced usage of prescription drugs, as was observed within the AIC cohort, could be associated with worse outcome. However, this is unlikely as the results also indicate, in addition to previous research findings showing likely health benefits with AIC, a concomitant reduction in the frequency of registered inpatient medical services (ICD-10:F chapter) for the AIC cohort. Future and prospective follow-up studies should address this issue by including drug utilization as well as clinical outcome and adverse event data.

The total cost for mental ill health including SRMD in Sweden is substantial and estimated to be in the range of EUR 10 billion [10]. However, the total direct cost estimate of all types of health care utilisation, including the cost of drugs, is only a small fraction - up to 20 % - of the total economical burden. The observed reduction in usage and cost of first-line drugs for patients referred to AIC was consistent and significant. Findings from the 180-days follow-up cohort in this study speculate that, an estimated value of EUR 137 per 1000 patients per day in costs of drugs could potentially be waived if AIC replaces conventional SRMD care. Although these figures are merely hypothetical and may seem rather small per patient, they could have significant impact over time considering a larger population suffering from SRMD. For example, employing the above estimates, a health insurer caring for one thousand SRMD patients could hypothetically save approximately EUR 50 000 per year in direct costs of first-line drugs alone by implementing AIC. Such impacts may be of interest to policy makers and health insurers alike. Nonetheless, such cost savings might not be perceived by e.g. patients and prescribing physicians, as there is a national insurance policy with an upper limit for what patients have to pay themselves per year for pharmaceuticals in Sweden.

The current consensus recommendations for SRMD rehabilitation include multi-modal care, which might include both pharmacological and physiological interventions [2, 12, 13]. However, SRMD rehabilitation may be complex both to formulate and evaluate, and the care process of deciding which treatments to combine in the rehabilitation of individual patients may not be straightforward. Antidepressants may be prescribed as pharmacotherapy option for treating depressive reactions in SRMD patients [13], but may also be prescribed in the management of anxiety and pain disorders [48, 49]. There is some evidence for the selective use of pharmacotherapy for SRMD, e.g. selective serotonin reuptake inhibitors in the treatment of patients suffering from post-traumatic stress disorders compared to placebo [50]. However, there remain crucial knowledge gaps in the evidence, e.g. whether or not to combine pharmacotherapy with other types of interventions including psychological therapies or for how long pharmacological treatment is clinically warranted. Clearly, one of the main challenges in the management of SRMD patients is to determine appropriate combinations of therapies that can be applied in the care of each individual patient.

The World Health Organization, through authoritative recommendations [18] and World Health Assembly resolutions [19], currently suggest member states to implement integrative care approaches within their national health systems. The strive for a salutogenic approach, where each patient encounter may be interpreted as a unique opportunity to collaboratively engage in a personalized patient-centred holistic healing process, may empower caregivers and patients alike in finding additional care strategies to pharmacotherapy [23, 42, 51] and lead to patients taking more responsibility for their own health [42, 51]. AIC has been suggested to facilitate new attitudes towards change and improved lifestyle habits that may improve patients’ health over time [52]. However, currently the knowledge base of the generalizability and patient preferences of AIC as well as the specific efficacy of individual and combinations of therapies and products are generally lacking. The academic underpinnings about the theoretical concepts of contemporary AIC may also need further clarifications to facilitate evidence informed communication and education. Nonetheless, taken together the emerging evidence for SRMD patients being referred to AIC seem to demonstrate health benefits such as increased health-related quality of life and self-rated health, together with reduced prescription drug utilisation and associated costs, which merit consideration for future research and evidence informed health sector reform.

### Limitations

Although Swedish health care registries are generally of very high standards with rigorous procedures, a problem is the inability to ensure proper and complete reporting from hospitals and health care units into the registries. This type of uncontrollable factors, e.g. under-reporting of co-morbid symptoms and disorders, including the level of accuracy in diagnostic criteria, may have methodological implications. Similarly, the selected SRMD diagnosis code ICD-10: F43 is rather wide, encompassing several different subgroups, each with unique aspects. However, such imprecisions can be considered to occur across all carers and patients and thus may be considered random noise. Specifically, the descriptive findings of concurrent overall disease load did not show any visually apparent differences at baseline between AIC and CC patients. A major methodological limitation was the lack of detailed information about SRMD duration, onset and severity of symptoms in the registry data and also that there were no specific details of the individual treatment plans in the registries for either CC or AIC. Further, using a retrospective cohort study design, which is mainly hypothesis generating, the utilisation of AIC and prescription of pharmaceuticals is likely based on patient and provider preferences with a risk for selection bias. Ideally, a prospective randomized clinical trial design is needed to properly address such concerns. Similarly, the reported differences in usage of drugs in this study might be due to other unknown factors and confounders, such as patient education or life-style choices, which were not possible to assess in the data or the matching procedure. While this may be true, the argument may be inapt for real-life decision-making, where patients can choose their type of care and the question arises which costs and benefits are associated with such choice. Based on the observed results within the AIC cohort over time it may be hypothesized that prescriptions and costs of first-line drugs decrease for SRMD patients that are referred to AIC and that benefits are likely. However, the generalizability of such hypothesis beyond the current setting is unknown despite that AIC can be found in many countries internationally. Multi-centre randomized clinical trials would be needed to investigate and examine such possibilities.

In the analysis, both DDD and cost outcomes data showed some positive skewness when investigated by Shapiro-Wilk and Kurtosis tests, albeit the skewness was minimal for the DDD. Hence, we principally maintained parametric testing procedures, alongside some non-parametric tests, in the analyses. Similarly, at 90 and 180 days before index there were some baseline differences in terms of DDD and cost data of two drug categories. Although different statistical modifications may be considered to adjust for such differences, it was not deemed necessary, as the primary interest was to explore the change scores within groups over time, and secondary to contrast the differences between the cohorts.

### Future research

Future prospective clinical trials in the area of conventional and integrative management of SRMDs are clearly warranted and should consider adverse events, tolerability of care as well as control for any additional interventions outside the health system, such as the use of non-prescription drugs and self-care measures alongside other clinical outcome measurements. Clinical investigations of integrative care have typically evaluated the specific efficacy of various therapies in isolation. In contrast, the comparative effectiveness of different packages of care under pragmatic “real life” health care situations is less well known. But such data increasingly represent the basis on which health technology assessment boards make recommendations. Given the prevalence and costs of SRMD and the emerging effects of integrative care approaches on patient quality of life and health economic measures, future studies may also consider investigating the dose and duration of contributing therapies, patient preferences, and determining factors that predict response to care. The application of pragmatic multi-centre clinical trial designs would facilitate generalizability across locations and settings.

## Conclusions

The findings of the current study suggests that there are different drug utilization patterns for SRMD patients referred to AIC or CC. Different management strategies between AIC and CC providers as well as different SRMD disease severities and preferences of patients referred to AIC and CC are hypothetical differentiating factors that may have influenced the observed drug outcomes over time. Additional studies including prospective and randomized clinical trials are warranted to determine if there is a causal link between referring patients to inpatient AIC and reduced utilization of first-line drugs thereafter.
